# Comparison of the antimicrobial activity of Ulmo honey from Chile and Manuka honey against methicillin-resistant Staphylococcus aureus, Escherichia coli and Pseudomonas aeruginosa. 

**DOI:** 10.1186/1472-6882-10-47

**Published:** 2010-09-02

**Authors:** Orla Sherlock, Anthony Dolan, Rahma Athman, Alice Power, Georgina Gethin, Seamus Cowman, Hilary Humphreys

**Affiliations:** 1 Department of Clinical Microbiology, Royal College of Surgeons in Ireland, Dublin, Ireland; 2 Research Centre, Faculty of Nursing and Midwifery, Royal College of Surgeons in Ireland, Dublin, Ireland; 3 Department of Microbiology, Beaumont Hospital, Dublin, Ireland; 4 Department of Applied Sciences, Dundalk Institute of Technology, Dundalk, Co. Louth, Ireland

## Abstract

**Background:**

Honey has previously been shown to have wound healing and antimicrobial properties, but this is dependent on the type of honey, geographical location and flower from which the final product is derived. We tested the antimicrobial activity of a Chilean honey made by *Apis mellifera *(honeybee) originating from the Ulmo tree (*Eucryphia cordifolia*), against selected strains of bacteria.

**Methods:**

Ulmo 90 honey was compared with manuka UMF^® ^25+ (Comvita^®^) honey and a laboratory synthesised (artificial) honey. An agar well diffusion assay and a 96 well minimum inhibitory concentration (MIC) spectrophotometric-based assay were used to assess antimicrobial activity against five strains of methicillin-resistant *Staphylococcus aureus *(MRSA), *Escherichia coli *and *Pseudomonas aeruginosa*.

**Results:**

Initial screening with the agar diffusion assay demonstrated that Ulmo 90 honey had greater antibacterial activity against all MRSA isolates tested than manuka honey and similar activity against *E. coli *and *P. aeruginosa*. The MIC assay, showed that a lower MIC was observed with Ulmo 90 honey (3.1% - 6.3% v/v) than with manuka honey (12.5% v/v) for all five MRSA isolates. For the *E. coli *and *Pseudomonas *strains equivalent MICs were observed (12.5% v/v). The MIC for artificial honey was 50% v/v. The minimum bactericidal concentration for all isolates tested for Ulmo 90 honey was identical to the MIC. Unlike manuka honey, Ulmo 90 honey activity is largely due to hydrogen peroxide production.

**Conclusions:**

Due to its high antimicrobial activity, Ulmo 90 may warrant further investigation as a possible alternative therapy for wound healing.

## Background

The use of honey as a traditional remedy for microbial infections dates back to ancient times [1]. The difference in antimicrobial potency among the different honeys can be more than 100-fold, depending on its geographical, seasonal and botanical source as well as through harvesting, processing and storage conditions [2]. The antimicrobial activity of honey is attributed largely to osmolarity, pH, hydrogen peroxide production and the presence of other phytochemical components e.g. methylglyoxal (MGO) [3]. *In vivo*, such activity may occur due to a synergistic relationship between any of these components rather than a single entity.

Manuka honey which originates from the manuka tree (*Leptospermum scoparium*) is sold as a therapeutic agent world wide. The presence of MGO in manuka honey contributes to its uniqueness and has been termed the unique manuka factor (UMF^®^). To date there are many publications reporting both *in vitro *and *in vivo *on the therapeutic properties of manuka honey, which have confirmed its activity against a wide range of medically important bacteria including methicillin-resistant *Staphylococcus aureus *(MRSA) [4,5]. As the potential role for honey as a topical agent to manage surgical site or wound infections is increasingly acknowledged [6], other honeys need to be assessed and evaluated. Newly identified honeys may have advantages over or similarities with manuka honey due to enhanced antimicrobial activity, local production (thus availability), and/or greater selectivity against medically important organisms [7].

In the current clinical environment, with the emergence of multi-drug resistant strains of bacteria, potential therapeutic agents to aid their control and eradication warrant investigation. Here we report an initial *in vitro *evaluation of honey originating from the Ulmo tree (*Eucryphia cordifolia*) native to Chile, compared with manuka and a laboratory synthesised honey.

## Methods

### Bacterial Strains

The antibacterial properties of three honeys were tested against seven bacterial isolates, i.e. three reference strains, *Escherichia coli *ATCC 35218, *Pseudomonas aeruginosa *ATCC 27853 and MRSA ATCC 43300 and four MRSA clinical isolates (two nasal, 01322 and 00745 and two surgical site/wound isolates, 00791 and 28965, from patients in an Irish hospital). The clinical isolates were identified as MRSA by routine laboratory methods; detection of staphylocoagulase and clumping factor (Staphaurex Plus, Remel, U.K.), and oxacillin resistance [by determining oxacillin minimum inhibitory concentrations (MIC)] using the MIC Evaluators system (Oxoid, U.K.).

### Honey samples

Three honey specimens were used in this study. Manuka honey with UMF^® ^25+ (Comvita^®^, New Zealand), Ulmo 90 honey (Rio San Pedro Ltd. Chile) and a laboratory synthesised (artificial) honey. The Ulmo 90 honey specimen was produced from the Ulmo tree (*Eucryphia cordifolia*), a large evergreen shrub, part of the temperate rain forests of southern Chile that flowers from the end of January until March. It had been analysed previously by Rio San Pedro Ltd. and 90% of the nectar source in this honey comes from the Ulmo tree with only very small proportions coming from other native Chilean rainforest plants http://www.riosanpedro.net/our_unique_ulmo_honey.html. The sample was a broad-based representative sample taken from a homogenised batch of 10 tonnes of 90% Ulmo honey, harvested between 24.03.08 and 04.04.08 and taken from various apiaries in the area of Lago Riñihue. The sample was not gamma-irradiated. Laboratory synthesised honey was prepared by weighing and dissolving 3.0 g sucrose, 15 g maltose, 80.1 g fructose and 67 g glucose (all supplied by Sigma-Aldrich, Ireland) in 34 ml sterile deionised water. The solution was heated briefly to 56°C in a water bath to aid dissolving. This formulation represents the four predominant sugars found in natural honey samples [8]. To prevent photodegradation of glucose oxidase found in honey, giving rise to hydrogen peroxide antimicrobial activity and to prevent the loss of hydrogen peroxide activity during analysis, all honey samples were stored at room temperature in the dark prior to testing and honey dilutions were prepared fresh daily prior to testing. A serial double dilution of honey (Ulmo, manuka or laboratory honey) was prepared aseptically for use in both the agar well diffusion and MIC assay from 50% to 0.02% v/v in nutrient broth (Oxoid, Fannin, Ireland). From the 50% (v/v) honey solution, 12 serial 1:1 dilutions were made, resulting in final concentrations of; 50%, 25%, 12.5%, 6.3%, 3.1%, 1.6%, 0.8%, 0.4%, 0.2%, 0.1%, 0.04%, and 0.02%. Hereafter, these solutions are referred to as 'test honey'.

### Well diffusion assay

A screening assay using well diffusion [9] was carried out with some minor modifications. Nutrient agar plates (Oxoid, U.K.) were inoculated by rubbing sterile cotton swabs that were dipped into bacterial suspensions (over night cultures grown at 37°C on nutrient agar and adjusted to 0.5 McFarland in sterile saline) over the entire surface of the plate. After inoculation 8.2 mm diameter wells were cut into the surface of the agar using a sterile cork borer. Eighty micro-litres of test honey, at each of the concentrations stated above, were added to each well. Plates were incubated at 37°C for 24 h. For *P. aeruginosa*, plates were incubated at 30°C. A diffusion control of methylene blue was used [10]. Zones of inhibition were measured using a Vernier caliper (Draper). The diameter of zones, including the diameter of the well, was recorded. Each assay was carried out in triplicate.

### Spectrophotometric assay for MIC determination

A previously described spectrophotometric assay for MIC determination [10] was performed. Minimum inhibitory concentrations were determined in sterile 96 well round bottomed polystyrene microtitre plates (Corning costar Ltd., NY). Ten μl of 0.5 McFarland standardised culture (prepared as described above) was added to 190 μl of test honey, at each of the concentrations stated above, in each well (six replicates per dilution, 12 dilutions tested). Control wells contained broth only (negative or sterility control) or bacteria and broth (positive control). Plates were incubated in the dark at 37°C with shaking at 150 rpm for 24 h. Data were analysed according to Patton *et al*., 2005 [10]. Briefly, the optical density was determined just prior to incubation (T0) and again after 24 h incubation (T24) at 600 nm. The OD for each replicate at T0 was subtracted from the OD for each replicate at T24. The adjusted OD of each control well was then assigned a value of 100% growth. The percent inhibition of growth was thus determined using the formula Percent Inhibition = 1- (OD test well/OD of corresponding control well) × 100. The MIC is reported as the lowest concentration of test material which results in 100% inhibition of growth of the test organism.

To establish if the antibacterial activity of the three honey samples was bacteriostatic or bactericidal [11], 10 μl from a well with each concentration of honey where bacterial growth was inhibited, were plated on to Colombia Blood Agar (CBA) (Oxoid) and incubated overnight. Plates with visible colony growth were considered to correspond to bacteriostatic honey activity while those with no growth were recorded as representing bactericidal honey activity.

### Hydrogen peroxide activity of honey

In order to determine if each of the honeys (Ulmo 90 and manuka) had non-peroxide antimicrobial activity, honey dilutions (50% - 1.6% v/v) were prepared in nutrient broth containing catalase (Sigma, C-40) at a final concentration of 0.2% w/v [12]. The assay was then conducted similar to the MIC determination above, using the spectrophotometric assay, with the exception that only MRSA isolate 0791 was used. Ten μl of 0.5 McFarland standardised culture of MRSA isolate 0791 was added to 190 μl of test honey in each well (five replicates per dilution). Control wells received broth and catalase only (negative or sterility control) or bacteria and broth and catalase (positive control). Plates were incubated in the dark at 37°C at 150 rpm for 24 h. After 24 h incubation, 10 μl from each well was inoculated on to a CBA plate. Plates were incubated at 37°C for 24 h and subsequently examined for growth.

### Results

Initial screening with the agar diffusion assay demonstrated that Ulmo 90 honey had superior antibacterial activity against all isolates compared with manuka honey. Table 1 outlines the antibacterial activity based on the zone of clearing that was produced. Ulmo 90 honey produces a large or equivalent antibacterial effect than manuka honey for each dilution when tested against the five MRSA isolates. In addition at lower concentrations, Ulmo honey continued to have an antibacterial effect for three of the five MRSA isolates. Comparable results against *E. coli *and *P. aeruginosa *were observed.

**Table 1:** Mean Zones of Inhibition (diameter mm including well (8.2 mm))

Concentration	50% v/v		25% v/v		12.5% v/v		6.3% v/v	
**Isolates**	**Ulmo**	**Manuka**	**Ulmo**	**Manuka**	**Ulmo**	**Manuka**	**Ulmo**	**Manuka**

MRSA ATCC 43300	30 (1.7)	24 (1.5)	26 (0.6)	19 (2.1)	18 (0.6)	13 (1.0)	10 (0.6)	-
MRSA 0791*	34 (1.5)	23 (1.2)	29 (1.7)	17 (1.7)	22 (2.1)	-	14 (2.5)	-
MRSA 28965*	24 (1.0)	17 (1.7)	19 (1.5)	15 (2.0)	-	-	-	-
MRSA 01322*	28 (5.8)	22 (1.0)	23 (4.2)	18 (0.6)	17 (2.9)	-	11 (2.0)	-
MRSA 0745*	23 (2.7)	20 (1.7)	19 (2.1)	13 (1.7)	11 (2.7)	-	-	-
*P. aeruginosa *ATCC 27853	14 (2.3)	16 (7.8)	11 (1.0)	14 (6.9)	-	-	-	-
*E. coli *ATCC 35218	14 (1.5)	15 (2.5)	11 (1.7)	12 (2.9)	-	-	-	-

* Clinical isolates

The antimicrobial activity of Ulmo honey in comparison to manuka honey against five MRSA strains, *E. coli *and *Pseudomonas aeruginosa*, assessed by agar well diffusion assay (n = 3). Where no value is given, no zone of inhibition was observed.

Standard deviation values (±) are in brackets

The MIC plate assay, a more sensitive measure of antimicrobial activity [10], showed that growth of all seven isolates was largely inhibited by both manuka honey and Ulmo 90 honey in comparison to the laboratory synthesised honey (Table 2). A lower MIC was observed for Ulmo 90 honey (3.1%-6.3% v/v) in comparison to manuka honey (12.5% v/v) for all five MRSA strains. Equivalent MICs were found for *E. coli *and *P. aeruginosa *for both honey types (12.5% v/v). The MIC for the laboratory synthesised honey for all strains was 50% v/v. The MIC for Ulmo 90 honey was also the minimum bactericidal concentration (MBC) for all isolates tested, with the exception of MRSA 01322 and 0745). For manuka and the laboratory honey the MBC was equivalent to the MIC.

**Table 2:** Minimum inhibitory concentration (MIC) and minimum bactericidal concentration (MBC) of Ulmo, manuka and laboratory synthesised honey necessary to inhibit 100% of the microbial growth *in vitro *expressed in % v/v solution (n = 3).

Isolate	Ulmo		Manuka		Laboratory
	**MIC**	**MBC**	**MIC**	**MBC**	**MIC & MBC**
	
MRSA ATCC 43300	6.3	6.3	12.5	12.5	50
MRSA 0791*	3.1	3.1	12.5	12.5	50
MRSA 28965*	6.3	6.3	12.5	12.5	50
MRSA 01322 *	3.1	6.3	12.5	12.5	50
MRSA 0745*	3.1	6.3	12.5	12.5	50
*P. aeruginosa *ATCC 27853	12.5	12.5	12.5	12.5	50
*E. coli *ATCC 35218	12.5	12.5	12.5	12.5	50

* Clinical isolates

Values are modal values and expressed as a percentage of undiluted honey.

The removal of hydrogen peroxide activity from Ulmo 90 honey reduced its antimicrobial activity. A 25% v/v solution of the Ulmo 90 had no detectable antibacterial activity when tested in the presence of catalase. In contrast, a 25% v/v solution of manuka maintained its antibacterial activity in the presence of catalase/absence of hydrogen peroxide.

## Discussion

The *in vitro *antibacterial activity of Ulmo 90 and manuka honey was evaluated and compared. Data obtained from the agar diffusion and spectrometric assays has demonstrated, for the first time, that Ulmo 90 honey exhibits a stronger peroxide attributable antimicrobial effect against five out of seven bacterial isolates tested compared with manuka honey. Using the agar diffusion method, on average, Ulmo honey displayed larger zones of inhibition against all MRSA strains. However, in some cases, large standard deviations were observed (Table 1). This may be accounted for by the method used to inoculate the bacteria on to the surface of the agar. Although this method has been used in previous studies [10], a more precise method may be to seed the agar with the test organism as described by Allen *et al*. (1991) [12].

A lower MIC was observed for Ulmo 90 honey (3.1% - 6.3% v/v) in comparison to manuka (12.5% v/v) for all five MRSA strains. Although this difference, which is one dilution, may not be significant. A previous report [13], showed that the MBC from Medihoney (contains manuka honey) against MRSA was 3% while ours was 3.1% v/v for Ulmo 90 for 3 of the 5 strains and 6.3% v/v for the other two. That previous report [13] proposed that there are differences in the susceptibility of strains of the same species, which we have confirmed for MRSA isolates. The MIC values for manuka honey may seem high (12.5%), especially when compared to Patton *et al*. (2006) [10], where the same spectrophotometric assay was used. That study used a less potent manuka honey (UMF 18+) with a resulting MIC of 6.25% v/v. However, the differences observed between that study and the current study may explain this anomaly, e.g. a different strain of *S. aureus *was used in that study.

The removal of hydrogen peroxide activity from Ulmo 90 was shown to have reduced its antimicrobial activity. A 25% v/v solution of the Ulmo 90 had no detectable antibacterial activity when tested in the presence of catalase, where previously a 3.1% v/v solution of Ulmo honey was both the MIC and MBC for MRSA strain 0791. This would suggest that bacterial inhibition in the previous experiments was mainly due to hydrogen peroxide generation. Although some activity was observed in Ulmo 90 at 50% v/v concentration, the same activity was seen in the laboratory synthesised honey, which may indicate that activity at this concentration may be due to other factors such as osmotic pressure or high sugar content. In contrast, while the MIC and MBC was affected, a 25% v/v solution of manuka displayed antibacterial activity in the presence of catalase i.e. this was the dilution at which both MIC and MBC was observed on the removal of peroxide activity. This finding was expected for manuka as it has been previously shown that its antibacterial activity is attributed to non-peroxide components such as MGO [12]. As catalase is present in body tissues, this may have an effect on the *in vivo *activity of hydrogen peroxide-dependent honeys. However the extent of this effect is not known.

Similar to other studies, this paper presents the findings of *in vitro *antibacterial activity of a honey against planktonic bacteria and therefore results cannot be extrapolated to the chronic wound environment. The chronic wound harbours up to four different wound pathogens [14] and indeed the presence of bacterial wound biofilms compound the difficulties in understanding and managing such an environment [13]. Within the biofilm, the characteristics of the bacteria change, so that biofilm-embedded bacteria are up to 1000 times more resistant to antibiotics than the 'planktonic' bacteria that are used to test antibiotic sensitivity [15]. The antibacterial nature of honey is dependent on various factors working either singularly or synergistically, the most salient of which are; hydrogen peroxide (produced by the glucose oxidase added to honey by bees), phenolic compounds, wound pH, pH of honey; osmotic pressure exerted by the honey, cleansing of the wound bed by the honey, level of exudate and the frequency of application. The degree to which any one of these contribute to *in vivo *antimicrobial efficacy has yet to be determined. However, a recent study examining the antimicrobial properties of honey *in vitro *found that hydrogen peroxide, MGO and an antimicrobial peptide, bee defensin-1, were distinct mechanisms involved in the bactericidal activity of honey [16]. In addition to its antimicrobial properties, the effects of honey on host cells may also play an important role in wound healing [17,18]. Therefore to focus solely on peroxide in honey limits our understanding of how honey may contribute to managing the bacterial wound bioburden.

## Conclusion

From the results contained in this report we conclude that, due to its high antimicrobial activity, Ulmo 90 may warrant further investigation into its use as a possible alternative therapy for wound healing.

## Competing interests

The authors declare that this study was supported in part by a Health Research Board Ireland Translational Research Grant (TRA/2006/04) and funding for consumables was received from Rio San Pedro Ltd., Chile, which manufactures Ulmo 90 honey.

## Authors' contributions

OS designed the study, analysed the data and drafted the manuscript. AD participated in study design, microbiological analysis and drafted the manuscript. RA and AP carried out testing of the various honey samples. GG, SC and HH conceived the study, secured funding and participated in manuscript preparation. All authors have read and approved

the final manuscript.

## Pre-publication history

The pre-publication history for this paper can be accessed here:

http://www.biomedcentral.com/1472-6882/10/47/prepub
